# Management of infrabony defects in mandibular molars in a patient with generalized aggressive periodontitis using autogenous bone graft from maxillary tuberosity

**DOI:** 10.4103/0972-124X.65443

**Published:** 2010

**Authors:** Sangeeta Singh

**Affiliations:** *320 Field Hospital, C/O 99APO-903 320, India*

**Keywords:** Autogenous bone graft, generalized aggressive periodontitis, infrabony defect

## Abstract

This clinical case report presents a technique utilizing autogenous cancellous bone from maxillary tuberosity to fill two infrabony defects distal to mandibular molars in a patient diagnosed with aggressive periodontitis. After debridement a 6 mm defect was present distal to mandibular right first molar and 6.5 mm defect was present distal to mandibular second molar of same side. Autogenous bone graft from maxillary tuberosity was placed in both the defects. There was a significant bony fill present six months post surgery and probing depth reduced by 7mm on both the sites.

## INTRODUCTION

Two techniques with the most successful documentation of periodontal regeneration are osseous grafting and guided tissue regeneration.[1] Traditional approach to correct periodontal pockets consisted of surgical debridement and resective procedures. These methods typically heal by repair forming a long junctional epithelium.[2]

Bone replacement grafts are the most widely used treatment options for the correction of periodontal osseous defects.[3] Bone replacement grafts include autografts, allografts, xenografts and alloplasts. Extra-oral autogenous grafts (iliac grafts) showed good results, however a common complication is root resorption.[4]

Intra-osseous defects grafted with autogenous grafts from intra-oral sites, especially maxillary tuberosity or healing extraction site, have demonstrated bony fill equal to that obtained from iliac grafts.[5,6]

## CASE REPORT

A 33-year-old male patient reported with the chief complaint of swelling and mobility in lower posterior teeth since six days. On examination there was a periodontal pocket of 7 mm and 8 mm on distal aspect of 46 and 47 respectively [Figure 1a and b]. Radiograph showed infrabony defects distal to 46 and 47 [Figure 1c]. The treatment plan was to perform an open flap debridement in 46, 47 region and utilize autogenous bone procured from maxillary tuberosity to fill the osseous defect.

**Figure 1a:**
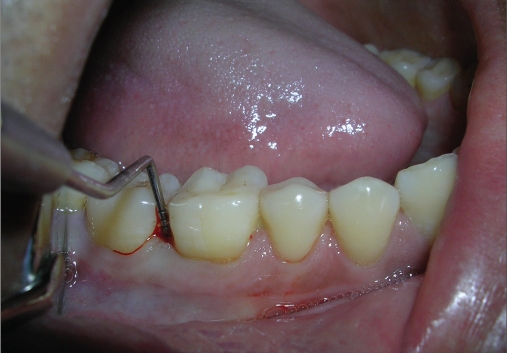
A periodontal pocket of 7 mm on distal aspect

**Figure 1b:**
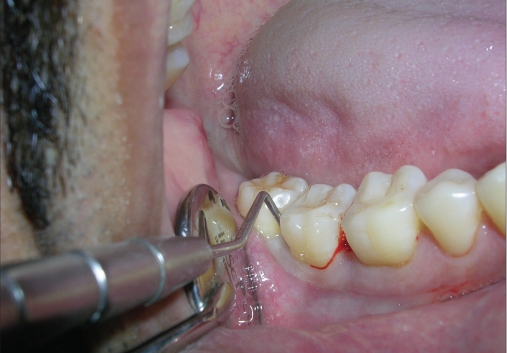
A periodontal pocket of 8 mm on distal aspect

**Figure 1c:**
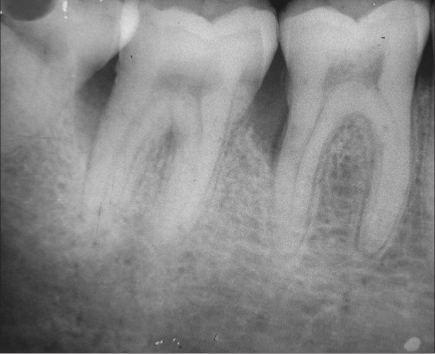
Radiograph showing infrabony defects distal to 46 and 47

### Surgical technique

A mucoperiosteal flap was raised from the 45-48 regions using simplified papilla preservation flap[7] to achieve maximum coverage of the grafted site. No vertical incisions were given and the flap was extended to one tooth on either side for adequate reflection [Figure 2a]. A thorough debridement was carried out and after complete removal of granulation tissue, 5.5 mm and 5 mm defects were present distal to 46 and 47 respectively [Figure 2b and c].

**Figure 2a:**
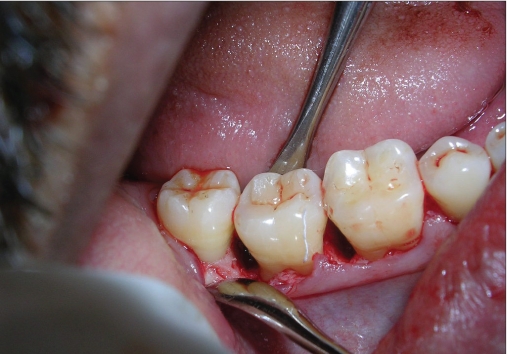
A flap extended to one tooth on either side for adequate reflection

**Figure 2b:**
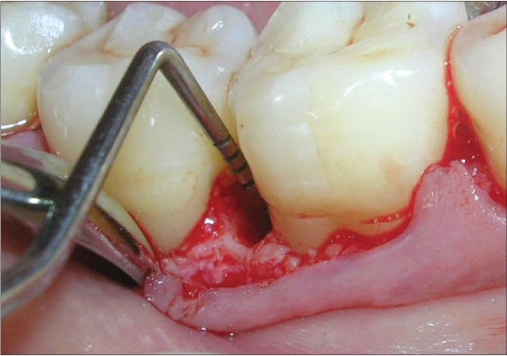
A defect of 7 mm distal to 46 seen on surgical exposure

**Figure 2c:**
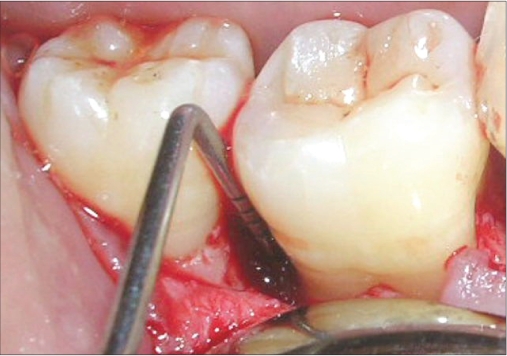
A defect of 8 mm distal to 47

**Figure 3:**
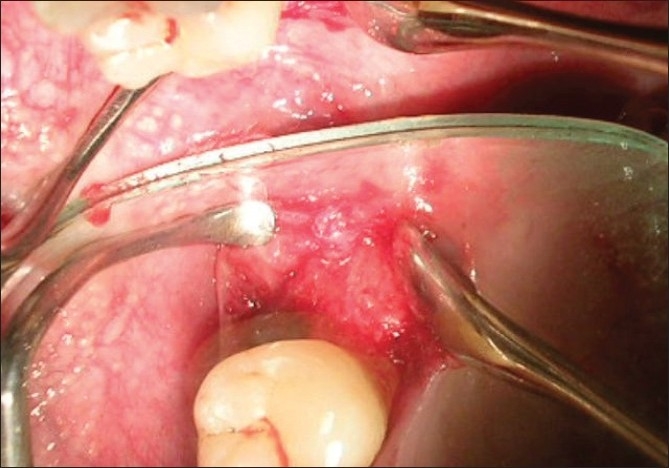
The right maxillary donor site

**Figure 4:**
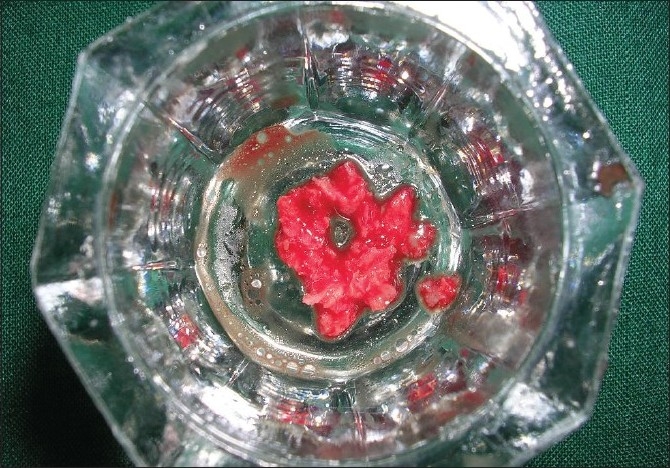
Autogenous cancellous bone chips collected from the donor site

**Figure 5:**
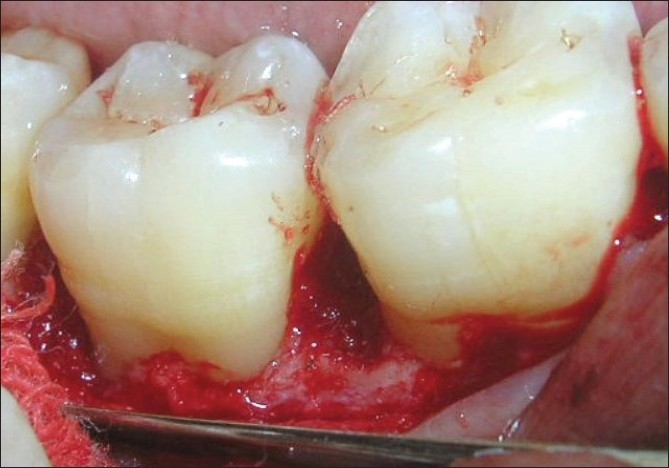
The graft used to fill up the defects distal to 46 and 47

The site selected for procurement of the autogenous graft was the right maxillary tuberosity. A mucoperiosteal flap was raised and site exposed with adequate reflection [Figure 3]. Autogenous chips were collected from the site [Figure 4] and placed into the defects [Figure 5]. The flaps were sutured with close approximation of both the flaps using interrupted sutures starting from the distal aspect of 47 and last suture placed between 45 and 46.

### Post surgical treatment and follow-up

The patient was instructed on proper plaque control in all areas except the quadrant of surgery where he was instructed not to brush. A 0.12% Chlorhexidine mouthwash was prescribed for rinsing twice daily. The sutures were removed 10 days after surgery and the patient was advised to continue the Chlorhexidine mouthwash for two weeks. After this he was advised to start mechanical plaque control in the operated quadrant using soft brush and roll technique.

The patient was put on regular recall at 1,3,6,9 and 12 months. The clinical picture had improved considerably at the time of three-month recall visit and there was no pain or bleeding from the sites. After nine months, the probing depth had reduced to 2 mm and 1.5 mm distal to 46 and 47 respectively [Figure 6a and b]. The radiograph showed good bony fill in the defects distal to 46 and 47 [Figure 6c]. Clinically, the sites appeared healthy, firm and with good adaptation to the underlying tissues.

**Figure 6a:**
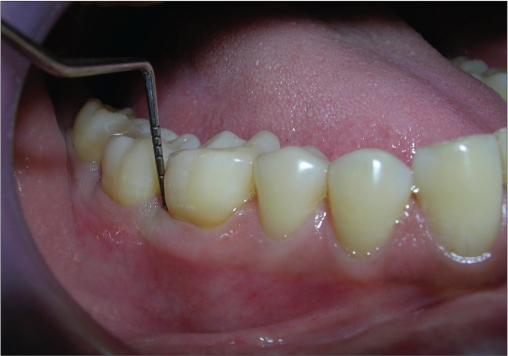
Postop probing depth of 3 mm distal to 46

**Figure 6b:**
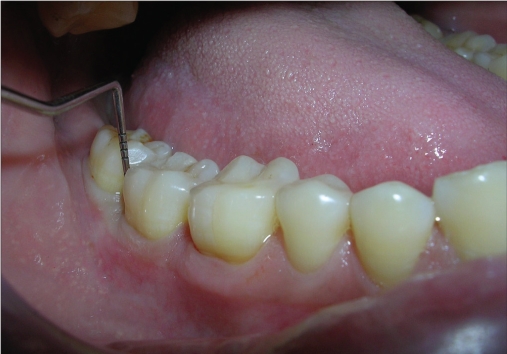
Postop probing depth of 2 mm distal to 47

**Figure 6c:**
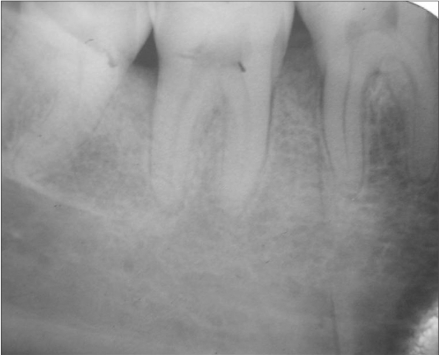
Postop IOPA showing significant bony fill distal to 46 and 47

## DISCUSSION

The aim of this case report was to present the use of autogenous bone in the management of intra osseous defects with excellent results. Periodontal pockets associated with intrabony lesions have been shown to be at higher risk of disease progression in patients who do not receive systematic periodontal therapy.[8] Autogenous grafts are considered the gold standard among graft materials and have excellent regenerative potential which has been proven earlier with histologic evidence.[9] The added benefit is the elimination of the risk of disease transmission and reduction in the cost of surgery by avoiding the use of alloplastic bone graft material.

Bone replacement grafts, including autogenous grafts from intraoral donor sites, allografts, xenografts, and alloplastic bone substitutes, are the most widely used treatment modalities for the regeneration of periodontal osseous defects. Studies suggest a favorable clinical outcome with the use of these materials in terms of improvements in periodontal probing depths, probing attachment gains, and bone fill. In terms of bone fill, most studies report more than 50% resolution of intrabony defects when treated with bone replacement grafts. However, histologic evidence of periodontal regeneration, including new bone, periodontal ligament, and cementum, has been reported only for autogenous bone grafts and demineralized freeze-dried bone allografts.[10]

The bone destruction that occurs in aggressive periodontitis is very rapid and requires immediate corrective measures to stop progression and further loss. The treatment plan should include oral hygiene instructions and reinforcement and evaluation of the patient's plaque control; supra- and subgin-gival scaling and root planing to remove microbial plaque and calculus; control of other local factors; occlusal therapy as necessary; periodontal surgery as necessary; and periodontal maintenance.

In this case the infrabony defects were narrow, three-walled and and the autogenous graft procured from tuberosity area was sufficient to fill the defects. The use of autogenous bone from maxillary tuberosity ensured predictable healing, excellent bone fill with a possibility of partial if not complete periodontal regeneration and elimination of risk of disease transmission.

## CONCLUSIONS

Within the limits of the present study it can be suggested that:

1. Autogenous bone grafting is still the best option for predictable regeneration in periodontal osseous defects.
2. The simplified papilla preservation technique ensures complete coverage of the grafted site and helps achieve excellent results.
